# CULTURALLY ADAPTING A TIMED ACTIVITY INTERVENTION FOR OLDER LATINOS WITH DEMENTIA AND THEIR CAREGIVERS

**DOI:** 10.1093/geroni/igz038.108

**Published:** 2019-11-08

**Authors:** Adriana G Perez

**Affiliations:** University of Pennsylvania School of Nursing, Philadelphia, Pennsylvania, United States

## Abstract

The number of Latinos living with Alzheimer’s Disease (AD) is expected to grow from 379,000 in 2012 to 3.5 million in 2060. The purpose of this study was to generate recommendations for meaningful approaches to culturally adapt a timed-activity intervention, Healthy Patterns, to promote quality of life in this population. Consistent with a descriptive qualitative approach, community-based focus groups were conducted among a purposive sample of older Latinos with AD and their caregivers. Seventeen dyads participated. Surface structure issues included: need for linguistically appropriate intervention sessions; partnerships with local, trusted community organizations. Deep structure themes, included: 1) embedding social support through group sessions; 2) culturally relevant activities; and 3) building individual and caregiver motivation for participation. This study gives “voice” to older Latinos and caregivers in adapting a timed-activity intervention. Results may inform the design and implementation of AD interventions, including recruitment and retention of older Latinos in clinical trials.

